# MiR-133a in Human Circulating Monocytes: A Potential Biomarker Associated with Postmenopausal Osteoporosis

**DOI:** 10.1371/journal.pone.0034641

**Published:** 2012-04-10

**Authors:** Yang Wang, Ling Li, Benjamin T. Moore, Xian-Hao Peng, Xiang Fang, Joan M. Lappe, Robert R. Recker, Peng Xiao

**Affiliations:** 1 Osteoporosis Research Center, School of Medicine, Creighton University, Omaha, Nebraska, United States of America; 2 Biostatistical Core, Office of Research and Compliance, Creighton University, Omaha, Nebraska, United States of America; Central China Normal University, China

## Abstract

**Background:**

Osteoporosis mainly occurs in postmenopausal women, which is characterized by low bone mineral density (BMD) due to unbalanced bone resorption by osteoclasts and formation by osteoblasts. Circulating monocytes play important roles in osteoclastogenesis by acting as osteoclast precursors and secreting osteoclastogenic factors, such as IL-1, IL-6 and TNF-α. MicroRNAs (miRNAs) have been implicated as important biomarkers in various diseases. The present study aimed to find significant miRNA biomarkers in human circulating monocytes underlying postmenopausal osteoporosis.

**Methodology/Principal Findings:**

We used ABI TaqMan® miRNA array followed by qRT-PCR validation in circulating monocytes to identify miRNA biomarkers in 10 high and 10 low BMD postmenopausal Caucasian women. MiR-133a was upregulated (*P*=0.007) in the low compared with the high BMD groups in the array analyses, which was also validated by qRT-PCR (*P*=0.044). We performed bioinformatic target gene analysis and found three potential osteoclast-related target genes, CXCL11, CXCR3 and SLC39A1. In addition, we performed Pearson correlation analyses between the expression levels of miR-133a and the three potential target genes in the 20 postmenopausal women. We did find negative correlations between miR-133a and all the three genes though not significant.

**Conclusions/Significance:**

This is the first *in vivo* miRNA expression analysis in human circulating monocytes to identify novel miRNA biomarkers underlying postmenopausal osteoporosis. Our results suggest that miR-133a in circulating monocytes is a potential biomarker for postmenopausal osteoporosis.

## Introduction

Tissue-specific expressed microRNAs (miRNAs) are short non-coding RNA molecules that regulate gene expression, generally by destabilizing mRNAs or suppressing translation. MiRNAs have been identified as important biomarkers and regulators in various human diseases such as cancer [1], [2], diabetes [3], [4] and myocardial disease [5]. In the bone area, many miRNAs regulate osteoblastogenesis [6]–[16]. However, very few miRNAs have been related to osteoclastogenesis [17], [18]. MiRNA miR-223 played an essential role in osteoclastogenesis in a mouse osteoclast precursor cell line [18]. MiR-146a inhibited osteoclastogenesis from human circulating mononuclear cells [17].

Circulating monocytes are important cells that participate in osteoclatogenesis by acting as osteoclast precursors [19]–[22] and secreting osteoclastogenesis-related factors, such as IL-1 (interleukin-1), IL-6 and TNF-α (tumor necrosis factor-alpha) [23]–[25]. In addition, human studies have found associations of gene expression levels in circulating monocytes and osteoporosis, such as ANXA2 (annexin A2) [26], STAT1 (signal transducer and activator of transcription 1) [27], CCR3 [chemokine (C-C motif) receptor 3], HDC (histidine decarboxylase), and GCR (glucocorticoid receptor) [28].

However, no study has been conducted to identify miRNA biomarkers in circulating monocytes associated with human osteoporosis *in vivo*. Our present study aimed to identify differentially expressed miRNAs in circulating monocytes isolated from postmenopausal Caucasian women with discordant bone mineral density (BMD) using ABI miRNA array technology followed by qRT-PCR (quantitative RT-PCR). We found the significance of miR-133a in human circulating monocytes associated with postmenopausal osteoporosis. Further bioinformatic analysis of miR-133a identified its potential target genes that may be important in osteoclastogenesis.

## Materials and Methods

### Human subjects and characteristics

The study was approved by the Institutional Review Board at Creighton University, and all the subjects signed informed-consent documents before entering the project. All the subjects were Caucasians of European origin recruited from the vicinity of Creighton University in Omaha, NE. The exclusion criteria were detailed in our previous mRNA expression profiling study on B cells isolated from postmenopausal Caucasians for different BMD status [29]. The information such as age, ethnicity, menstrual status, medication history, and disease history was obtained via questionnaire. We recruited 20 unrelated postmenopausal Caucasian women, 10 with high BMD (spine or hip Z-score>0.84) and 10 with low BMD (spine or hip Z-score<−0.84). The high and low BMD groups are the top and bottom 20% BMD distributions of the age-, sex- and ethnicity-matched population. BMD (g/cm^2^) for the lumbar spine (L1-4) and total hip (femoral neck, trochanter, and intertrochanteric region) were measured by Hologic 4500A dual energy X-ray absorptiometry (DXA) scanners (Hologic Inc., Bedford, MA). The machine was calibrated daily. The measurement precision as reflected by the coefficient of variation (CV) was 0.9% and 1.4% for spine and hip BMD, respectively. Postmenopausal status was defined as the date of the last menses followed by at least 12 months of no menses. All the study subjects were aged 57–68. The detailed characteristics of the study subjects are summarized in Table 1.

**Table 1:** Characteristics of the study subjects.

Traits	High BMD(n=10)	Low BMD(n=10)	*P* Value
Age (yrs)	63.6±3.2	61.6±2.6	0.15
Height (cm)	159.2±3.2	163.6±4.7	0.03
Weight (kg)	76.7±7.9	72.9±17.6	0.55
Spine BMD (g/cm^2^)	1.128±0.058	0.826±0.069	<0.001
Spine Z-Score	2.24±0.59	−0.63±0.64	<0.001
Hip BMD (g/cm^2^)	1.057±0.101	0.725±0.045	<0.001
Hip Z-Score	1.94±0.99	−1.04±0.45	<0.001

Note: The data are mean ±SD.

As shown in Table 1, both hip and spine BMD were significantly different between the high and low BMD groups. For age, weight and height traits, only height showed marginal difference between the two BMD groups. However, height only demonstrated a very small effect on quantitative BMD variations [30]. Moreover, in this study, BMD was classified as a quality trait into two categories, the low and the high BMD. Therefore, the effect of height on BMD can be ignored in this study.

### Monocyte isolation

Blood mononuclear cells (MNCs) from 70 ml peripheral blood from each study subject were separated by density gradients with UNI-SEP tubes containing a solution of 5.6% polysucrose and 9.6% sodium metrizoate with a density of 1.077 g/ml (Novamed, Jerusalem, Israel). Monocytes were isolated by a negative isolation kit, Dynabeads® Untouched™ Human Monocytes (Dynal Biotech, Lake Success, NY, USA), which contains a cocktail of CD2, CD7, CD16, CD19, CD56 and CD235a antibodies to deplete T cells, B cells, natural killer cells, erythrocytes and granulocytes, leaving monocytes naive and free of the surface-bound antibody and beads. The purity of isolated monocytes was assessed by flow cytometry with fluorescence labeled antibodies CD19-PE and CD45-FITC (BD Biosciences, San Jose, CA USA), and the average purity is about 85% with 3% deviation.

### Total RNA extraction

The *mir*Vana miRNA Isolation Kit (Ambion, Austin, Texas, USA) was used to extract total RNA including miRNAs from each cell sample following the manufacturer's protocol. Total RNA concentration and integrity were evaluated by an Agilent 2100 Bioanalyzer (Agilent, Palo Alto, CA, USA). Each RNA sample has a high quality with an excellent integrity number >9.0.

### MiRNA array procedures

We used TaqMan® Human MicroRNA Array v1.0 (Applied Biosystems, Foster City, CA, USA) to perform miRNA expression profiling for each RNA sample. Each array covers 365 human miRNAs and endogenous controls RNU48 and RNU44. First, TaqMan miRNA Multiplex Reverse Transcription Kit (Applied Biosystems) was used for the RT reaction. For each RNA sample, the RT reaction was performed in a 63 µl reaction system including 1.8 µl 100 mM dNTPs, 18 µl Reverse Transcriptase (50 U/ml), 9 µl 10× RT Buffer, 1.13 µl RNase Inhibitor (20 U/µl), 16 µl sample RNA, and 17.08 µl nuclease-free water. The reaction conditions were as follows: 30 min at 16°C, 30 min at 42°C, and 5 min at 85°C. After that, we mixed 450 µl diluted RT reaction product (diluted 62.5-fold) with 450 µl TaqMan Universal PCR Master Mix (ABI) and loaded 100 µl real-time PCR reaction mix into each port of the array card (8 ports/card). The real-time qRT-PCR for each array was carried out on an Applied BioSystems 7900HT Fast Real-time PCR System with the following reaction conditions: 2 min at 50°C, 10 min at 95°C, 40 cycles of 15 sec at 95°C plus 1 min at 60°C. For each array card, there was only one probe for each target miRNA.

In the miRNA array data analysis, the raw expression level was determined by the cycle number at which the reaction crossed a predetermined cycle threshold (CT) as identified for each miRNA probe. The relative quantity (RQ) of each miRNA for each sample is determined by 2^−ΔΔCT^, where ΔCT=(CT_Target miRNA_−CT_endogenous control RNU48_) and ΔΔCT=(ΔCT−average ΔCT of all the samples). The RQ data were used for student's *t* test to identify differentially expressed miRNAs between the high and the low BMD groups.

### qRT-PCR for miRNAs

To correct for the multiple-testing comparison and eliminate false positive results in the miRNA array analysis, we conducted qRT-PCR among the same 20 RNA samples to further validate the identified significant miRNAs in the array analysis. Two-step qRT-PCR was used to confirm the differentially expressed miRNAs. The first step is RT of cDNA and the second step is real-time quantitative PCR. All the reagents are provided by Applied Biosystems. The RT reaction was performed in a 15 µl volume, containing 1.5 µl Taqman RT Buffer (10×), 0.15 µl 100 mM dNTPs (100 mM), 1.0 µl Reverse Transcriptase, 0.19 µl RNase inhibitor (20 U/µl), 3.0 µl specific miRNA primer, 100 ng total RNA, and nuclease-free water to make the final volume 15 µl. The real-time quantitative PCR was performed in a 20 µl reaction volume using standard protocols on the Applied Biosystems 7900HT System. Briefly, 2.5 µl cDNA was mixed with 10.0 µl TaqMan universal PCR master mix (2×), 1.0 µl TaqMan miRNA assay and 6.5 µl nuclease-free water. The reaction conditions were the same as the above real-time PCR in the array experiments. For each RNA sample, the target miRNA and RNU48 reactions were run as triplicates in the same plate. The RQ of each miRNA for each sample is determined by 2^−ΔΔCT^, where ΔCT=(average of triplicate CT_Target miRNA_−average of triplicate CT_endogenous control RNU48_) and ΔΔCT=(ΔCT−average ΔCT of all the samples). The RQ data were used for student's *t* test between the two groups.

### Target gene prediction and verification

We conducted bioinformatic sequence analysis of each significant miRNA to identify potential target genes [31]. MiRNAs normally repress gene expression by base pairing at complementarity sites mainly but not exclusively in the 3′-untraslated region (3′-UTR) of the target mRNAs [32], [33]. The currently available miRNA target gene databases are all limited in the 3′-UTR analyses. We used both miRDB (http://www.miRDB.org/) and TargetScan (http://www.targetscan.org/) databases to predict target genes by searching for the presence of conserved 8-mer and 7-mer sites in their 3′-UTRs that match the seed region of each significant miRNA [34]. In addition, we also conducted qRT-PCR for the potential target genes of the significant miRNA among the same 20 RNA samples. Similar to miRNA qRT-PCR, the mRNA qRT-PCR was also composed of RT and real-time qPCR. The first step is RT of cDNA and the second step is real-time quantitative PCR. The RT and qPCR were in 100 µl and 25 µl volumes, respectively, following the company's standard protocols (Applied Biosystems). For each RNA sample, the target mRNA and internal control β-actin were run as triplicates in the same plate. We used the same calculation for RQ 2^−ΔΔCT^ as we did for miRNA qRT-PCR and performed student's *t* test between the two groups.

## Results

### MiRNA array analyses

**Figure 1:**
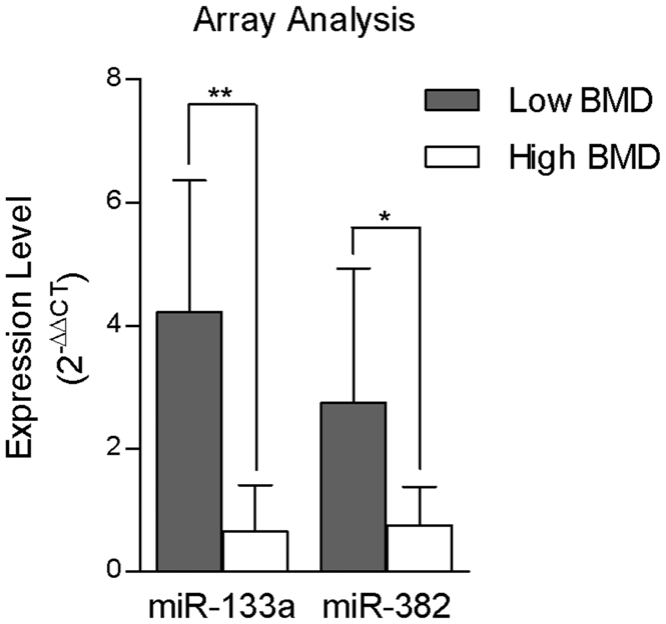
Expression levels (2^−ΔΔCT^) of significant miRNAs measured by array analysis in circulating monocytes in the low and high BMD groups (**: P<0.01; *: P<0.05).

Among the 365 miRNAs in the array, the expression of many miRNAs were missing among the 20 study samples, probably due to tissue-specific expression or extremely low expression. To obtain enough power, we selected miRNAs that were expressed in at least 5 samples in each BMD group for the analyses. According to this criterion, 156 qualified miRNAs (Table S1) were subject to the statistical analyses and two miRNAs, miR-133a and miR-382, showed significant upregulation in the low BMD group compared with the high BMD group (Figure 1). Specifically, miR-133a displayed a fold change of 6.48 between the low and high BMD groups as mean ± SD (4.21±2.15 vs. 0.65±0.75, P=0.007), and miR-382 showed a fold change of 3.65 between the low and high BMD groups (2.74±2.18 vs. 0.75±0.63, P=0.027).

### qRT-PCR for miRNAs

**Figure 2:**
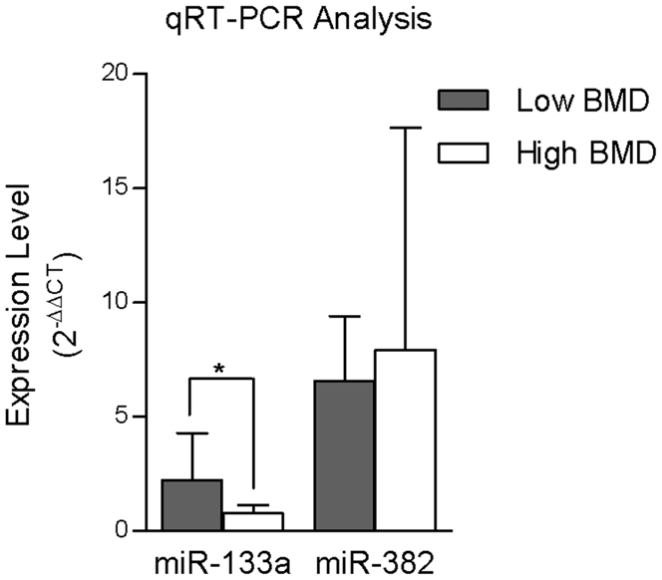
Expression levels (2^−ΔΔCT^) of miR-133a and miR-382 measured by qRT-PCR analysis in circulating monocytes in the low and high BMD groups (*: P<0.05).

We further performed qRT-PCR to validate the differential expression of miR-133a and miR-382. However, only the upregulation of miR-133a in monocytes in the low vs. the high BMD group (2.21±2.08 vs. 0.76±0.37) was validated by qRT-PCR (P=0.044). The difference in expression of miR-382 in monocytes in the low vs. the high BMD group (6.56±2.84 vs. 7.93±9.73) was not significant (P=0.67) (Figure 2).

### Target gene prediction and verification

Our bioinformatic sequence analyses identified 226 potential target genes that are overlapped in both miRDB and TargetScan databases. By searching relevant references for all the 226 genes, we found that three of them are related to osteoclastogenesis, CXCL11 [chemokine (C-X-C motif) ligand 11], CXCR3 [chemokine (C-X-C motif) receptor 3], and SLC39A1 [solute carrier family (zinc transporter), member 1]. Table 2 demonstrates the specific putative binding sites of miR-133a in the 3′ UTRs of the three genes. We conducted qRT-PCR analyses for all the three genes among the same 20 study samples and did not find significant differential expression. In addition, we performed correlation analysis of the expression levels of miR-133a and each gene. All three genes did demonstrate negative correlation with miR-133a, although they were not significant (P>0.05) (Table 3).

**Table 2:** Putative binding sites of miR-133a in predicted target genes in humans.

Target Gene	3′ UTRPosition	Consequential PairingTarget gene binding region (top) and miR-133a sequence (bottom)
		5′...AAAGGUGGGUGAAA**GGACCAA**A...
CXCL11	171–177	**|||||||**
		3′ GUCGACCAACUUCC**CCUGGUU**U
		5′...AAACAAGAUCGUCA**GGACCAA**A...
CXCR3	444–450	**|||||||**
		3′ GUCGACCAACUUCC**CCUGGUU**U
		5′...AAGGGAAAUACUGA**GGACCAA**A...
SLC39A1	107–113	**|||||||**
		3′ GUCGACCAACUUCC**CCUGGUU**U

**Table 3:** Correlation of expression levels (2^−ΔΔCT^) of miR-133a with those of three potential target genes and the ratio of expression levels of each gene in the high and low BMD groups, as measured by qRT-PCR.

Gene	Fold Change (H/L)	Correlation Coefficient	P Value
CXCL11	2.70	−0.04	0.88
CXCR3	3.02	−0.22	0.36
SLC39A1	1.33	−0.02	0.94

Note: H/L: high/low BMD groups.

## Discussion

In this study, we aimed to identify important miRNAs in human circulating monocytes associated with discordant BMD status in postmenopausal Caucasian women. We found significant upregulation of miR-133a in the low BMD group in both the array and the qRT-PCR analyses.

Human mature miR-133a is encoded by two genes: MIR133A1 for miR-133a1 at 18q11.2 (194,036,59–194,077,46 bp) and MIR133A2 for miR-133a2 at 20q13.33 (611,601,19–611,642,20 bp). Both genes encode different pre-mature miRNAs but generate the same mature miR-133a sequence. Interestingly, human genetic studies also found the association of 18q11.2 to osteoporosis-related traits [35] and linkage of 20q13 to bone phenotypes [36]–[38]. In humans, there are two types of miR-133 miRNA isoforms, miR-133a and miR-133b, with one base difference (g-a) in the last nucleotide at the 3′ end (miRBase: http://www.mirbase.org/). The ABI miRNA array used in this study includes both miR-133a and miR-133b probes. Interestingly, miR-133b is marginally unregulated in the low vs. the high BMD groups (1.51±0.67 vs. 0.95±0.63, P=0.08). In addition, we detected miR-133a expression levels in circulating B cells from the same 20 high or low BMD postmenopausal women. Circulating B cells were isolated by Dynabeads® CD19 (Pan B) (Dynal Biotech). However, miR-133a was not differentially expressed in B cells between the high and the low BMD groups (P=0.49). Therefore, miR-133a is most likely to be a monocyte specific biomarker underlying postmenopausal osteoporosis.

Many studies demonstrated that miR-133 and 133a are important in the development of muscle, such as skeletal muscle [39]–[43], and heart/cardiovascular muscle [44]–[47]. In bone, particularly, miR-133 and 133a have been found to regulate osteoblastogenesis by targeting and regulating Runx2 expression [6], [48]. A recent study also demonstrated that miR-133a was upregulated in osteoblast-like periodontal ligament stem cells treated with ibandronate, a nitrogen-containing bisphosphonate that inhibits bone resorption and is widely used to treat osteoporosis [49]. However, our study for the first time showed the association of miR-133a expression levels in circulating monocytes, the osteoclast precursors, with postmenopausal osteoporosis.

To further predict what genes are targeted and regulated by miR-133a in monocytes in bone metabolism, we used two miRNA target gene predicting databases (miRDB and TargetScan) [50]–[55]. Our bioinformatic sequence analyses and reference searching identified three potential target genes of miR-133a related to the inhibition of osteoclastogenesis, which are CXCL11, CXCR3, and SLC39A1 (Table 2). CXCL11 is a small cytokine of the CXC chemokine family. CXCL11 has been shown to inhibit osteoclast differentiation of CD14+ monocytes [56]. CXCR3 is a Gα_i_ protein-coupled receptor in the CXC chemokine receptor family. CXCR3 expression significantly decreased during osteoclast differentiation [57]. The SLC39A1 gene encodes zinc transporter 1 (ZIP1). Zinc deficiency has been correlated with reduction of bone growth and development of osteoporosis [58], [59]. SLC39A1 has been detected in osteoclasts and inhibited osteoclastogenesis and osteoclast function through zinc uptake [60].

**Figure 3:**
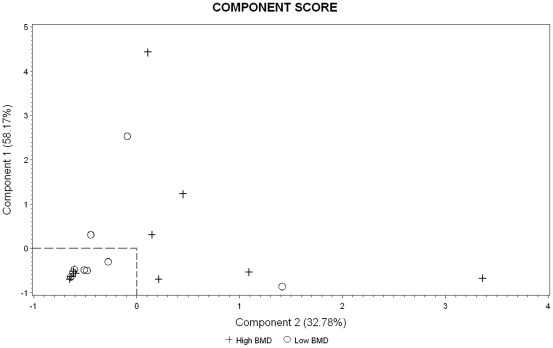
PCA of the expression levels of the three potential target genes measured by qRT-PCR in the 10 high and 10 low BMD subjects. In each axis, “0" represents the average level of each principle component.

All three genes did show negative correlation with miR-133a, though not significant (Table 3). Since one single miRNA normally regulates the expression of hundreds of genes, the regulatory effect of each gene may be small. Osteoporosis is a complex disease and regulated by multiple genes [61]. To investigate the combined effects, we also utilized a principle component analysis (PCA) to analyze the qRT-PCR observations from all three potential target genes. The PCA was performed using the correlation matrix. The analysis reduced the original data into two principle components (PC) that account for 58.17% and 32.78% of the total variance, respectively. A plot of the second PC against the first PC in the 10 high and 10 low BMD subjects is shown in Figure 3. We can see that both PCs are above the average levels in 6 out of 10 high BMD subjects and below the average levels in 7 out of 10 low BMD subjects, which means that the three genes are systematically upregulated in the high vs. low BMD groups. This result is consistently correlated with the downregulation of miR-133a in the high vs. low BMD groups.

There are some limitations for this study. In the miRNA array data, we performed the statistical tests for the 156 miRNAs according to expression data available in at least five samples in each group, and used raw *P* values but not adjusted *P* values for multiple tests. Actually, we did not find significant miRNAs after multiple testing adjustments by Bonferroni. However, the significant qRT-PCR *P* value confirmed the differential expression of miR-133a in the array analyses. The qRT-PCR validation largely solved the multiple testing problem. The sample size of 20 is relatively small. However, it is enough for an initial biomarker screening according to our previous [29], [62] and other [63], [64] gene expression profiling studies. Moreover, we are also planning to validate the miR-133a biomarker in a larger independent population. We did not find significant correlations of miR-133a and selected potential target genes. First, it may be due to the limited sample size to get enough power to detect the significant correlation. Second, many miRNAs inhibit gene expression by only suppressing protein translation instead of mRNA degradation [32], [33]. Third, there may be other unknown target genes of miR-133a in circulating monocytes that affect osteoclastogenesis. Therefore, we will test the current and potential novel target gene expression and correlation with miR-133a in a bigger population at both mRNA and protein levels in the future.

In summary, this is an initial miRNA expression study to indentify miRNA biomarkers in human circulating monocytes underlying postmenopausal osteoporosis. Our study suggested that miR-133a is a potential miRNA biomarker and/or regulatory element in circulating monocytes for postmenopausal osteoporosis.

## Supporting Information

Table S1 MiRNA array results of 156 miRNAs that were expressed in at least 5 samples in each BMD group. *: the ratio of the mean expression values between the low and high BMD groups.
(DOC)
