# Supplementary material for: MiR-133a in Human Circulating Monocytes: A Potential Biomarker Associated with Postmenopausal Osteoporosis
Source: PLoS One. 2012 Apr 10;7(4):e34641. doi: 10.1371/journal.pone.0034641 (PMC3323546; doi:10.1371/journal.pone.0034641)
Supplement: Table S1 — MiRNA array results of 156 miRNAs that were expressed in at least 5 samples in each BMD group. *: the ratio of the mean expression values between the low and high BMD groups. (DOC) [file pone.0034641.s001.doc]

| miRNA | Fold (L/H) * | P value |
| --- | --- | --- |
| miR-133a | 6.45 | 0.007 |
| miR-382 | 3.66 | 0.027 |
| miR-27b | 2.44 | 0.054 |
| miR-422a | 2.34 | 0.065 |
| miR-151 | 1.67 | 0.076 |
| miR-152 | 1.44 | 0.076 |
| miR-133b | 1.58 | 0.083 |
| miR-1 | 10.29 | 0.085 |
| miR-126-4373269 | 2.12 | 0.092 |
| let-7a | 2.31 | 0.095 |
| miR-24 | 1.46 | 0.098 |
| miR-221 | 2.47 | 0.108 |
| miR-137 | 2.17 | 0.113 |
| let-7b | 2.28 | 0.120 |
| miR-222 | 1.35 | 0.124 |
| miR-210 | 1.75 | 0.141 |
| miR-132 | 1.57 | 0.154 |
| miR-186 | 1.41 | 0.160 |
| miR-335 | 2.57 | 0.166 |
| miR-425 | 1.35 | 0.170 |
| miR-148b | 1.63 | 0.177 |
| miR-26a | 1.51 | 0.182 |
| miR-425-5p | 1.66 | 0.192 |
| miR-224 | 2.26 | 0.203 |
| miR-330 | 1.50 | 0.205 |
| miR-485-3p | 2.07 | 0.207 |
| miR-103 | 1.83 | 0.207 |
| miR-345 | 1.45 | 0.217 |
| miR-296 | 1.83 | 0.218 |
| miR-31 | 2.74 | 0.220 |
| miR-200c | 1.98 | 0.226 |
| miR-25 | 1.55 | 0.234 |
| miR-502 | 1.43 | 0.234 |
| miR-532 | 1.55 | 0.245 |
| miR-486 | 1.54 | 0.246 |
| miR-660 | 1.55 | 0.248 |
| miR-30a-5p | 1.53 | 0.254 |
| miR-134 | 2.30 | 0.256 |
| miR-199a | 1.67 | 0.261 |
| miR-361 | 1.83 | 0.261 |
| miR-30b | 1.47 | 0.268 |
| miR-30d | 1.54 | 0.271 |
| miR-182 | 1.88 | 0.278 |
| miR-423 | 1.82 | 0.282 |
| miR-142-5p | 1.51 | 0.283 |
| miR-30a-3p | 1.50 | 0.283 |
| miR-340 | 1.49 | 0.294 |
| miR-146b | 1.94 | 0.295 |
| miR-491 | 1.37 | 0.300 |
| miR-125a | 2.30 | 0.301 |
| miR-432 | 1.78 | 0.306 |
| miR-92 | 1.46 | 0.306 |
| miR-26b | 1.49 | 0.308 |
| miR-127 | 1.86 | 0.310 |
| miR-99a | 1.41 | 0.310 |
| miR-429 | 0.66 | 0.312 |
| let-7f | 1.57 | 0.314 |
| miR-126-4378064 | 2.05 | 0.324 |
| miR-324-5p | 1.47 | 0.328 |
| miR-155 | 6.51 | 0.334 |
| miR-650 | 0.65 | 0.348 |
| miR-223 | 1.28 | 0.349 |
| miR-191 | 1.80 | 0.353 |
| miR-17-5p | 1.40 | 0.353 |
| miR-487b | 2.03 | 0.358 |
| miR-28 | 1.55 | 0.360 |
| miR-422b | 1.50 | 0.360 |
| miR-200b | 1.54 | 0.362 |
| miR-19b | 1.19 | 0.370 |
| miR-629 | 1.29 | 0.378 |
| miR-365 | 1.40 | 0.378 |
| miR-98 | 1.84 | 0.382 |
| miR-545 | 1.95 | 0.382 |
| miR-148a | 1.40 | 0.385 |
| miR-550 | 1.30 | 0.386 |
| miR-181c | 1.36 | 0.397 |
| miR-192 | 1.72 | 0.399 |
| miR-30e-3p | 1.41 | 0.401 |
| miR-21 | 1.27 | 0.401 |
| miR-130a | 1.76 | 0.404 |
| miR-23b | 0.67 | 0.408 |
| miR-146a | 1.42 | 0.408 |
| miR-125b | 1.48 | 0.409 |
| miR-30c | 1.27 | 0.409 |
| miR-107 | 2.42 | 0.417 |
| miR-181b | 1.24 | 0.429 |
| miR-181d | 1.36 | 0.434 |
| miR-9-4378074 | 1.34 | 0.438 |
| miR-101 | 1.50 | 0.438 |
| miR-196b | 1.24 | 0.439 |
| miR-501 | 1.35 | 0.443 |
| miR-410 | 1.38 | 0.449 |
| miR-193a | 1.33 | 0.450 |
| miR-500 | 1.37 | 0.453 |
| miR-27a | 1.33 | 0.457 |
| miR-213 | 0.74 | 0.458 |
| miR-374 | 1.33 | 0.459 |
| miR-93 | 1.27 | 0.462 |
| miR-339 | 1.45 | 0.463 |
| miR-199b | 1.43 | 0.467 |
| miR-320 | 1.43 | 0.474 |
| let-7d | 1.33 | 0.475 |
| miR-19a | 1.18 | 0.477 |
| miR-20a | 1.21 | 0.482 |
| miR-301 | 1.55 | 0.498 |
| miR-99b | 1.33 | 0.502 |
| miR-100 | 1.25 | 0.513 |
| miR-362 | 1.21 | 0.516 |
| miR-618 | 0.68 | 0.525 |
| miR-484 | 1.10 | 0.527 |
| miR-378 | 1.23 | 0.545 |
| miR-376a | 1.21 | 0.568 |
| miR-328 | 1.23 | 0.607 |
| miR-195 | 1.20 | 0.608 |
| miR-149 | 1.29 | 0.613 |
| miR-145 | 1.35 | 0.615 |
| miR-7 | 0.78 | 0.618 |
| miR-18a | 0.72 | 0.622 |
| miR-449 | 1.43 | 0.623 |
| miR-15a | 0.84 | 0.624 |
| miR-197 | 1.14 | 0.628 |
| miR-15b | 1.25 | 0.632 |
| miR-218 | 0.66 | 0.634 |
| miR-331 | 1.14 | 0.647 |
| miR-142-3p | 1.26 | 0.649 |
| miR-342 | 1.11 | 0.650 |
| let-7c | 1.23 | 0.666 |
| miR-338 | 0.84 | 0.684 |
| miR-326 | 1.31 | 0.707 |
| miR-141 | 0.79 | 0.711 |
| miR-32 | 1.16 | 0.717 |
| miR-22 | 1.18 | 0.729 |
| miR-424 | 0.83 | 0.742 |
| miR-20b | 1.09 | 0.750 |
| miR-130b | 1.15 | 0.754 |
| miR-95 | 1.25 | 0.761 |
| miR-659 | 0.72 | 0.762 |
| miR-9-4373285 | 1.12 | 0.766 |
| miR-324-3p | 1.08 | 0.776 |
| miR-194 | 1.15 | 0.779 |
| miR-200a | 1.12 | 0.800 |
| miR-594 | 0.79 | 0.810 |
| miR-16 | 1.05 | 0.815 |
| miR-30e-5p | 1.10 | 0.821 |
| miR-565 | 1.15 | 0.825 |
| miR-196a | 1.12 | 0.830 |
| miR-140 | 1.06 | 0.832 |
| miR-29a | 1.09 | 0.852 |
| miR-23a | 1.06 | 0.868 |
| miR-29c | 1.07 | 0.869 |
| miR-433 | 0.92 | 0.880 |
| miR-106b | 1.04 | 0.921 |
| miR-10a | 1.07 | 0.932 |
| miR-17-3p | 1.04 | 0.937 |
| let-7g | 1.02 | 0.968 |
| miR-411 | 1.01 | 0.993 |
